# Identification of Novel Immunoregulatory Molecules in Human Thymic Regulatory CD4^+^CD25^+^ T Cells by Phage Display

**DOI:** 10.1371/journal.pone.0021702

**Published:** 2011-08-01

**Authors:** Georgia Porto, Ricardo J. Giordano, Luciana C. Marti, Beatriz Stolf, Renata Pasqualini, Wadih Arap, Jorge Kalil, Verônica Coelho

**Affiliations:** 1 Heart Institute, Instituto do Coração (InCor), School of Medicine, University of São Paulo, São Paulo, Brazil; 2 Division of Clinical Immunology and Allergy, University of São Paulo, São Paulo, Brazil; 3 Institute for Investigation in Immunology, National Institute of Science and Technology (iii-INCT), São Paulo, Brazil; 4 David H. Koch Center, MD Anderson Cancer Center, The University of Texas, Houston, Texas, United States of America; 5 Instituto Israelita de Ensino e Pesquisa Albert Einstein, São Paulo, Brazil; 6 Institute of Biomedical Sciences, University of São Paulo, São Paulo, Brazil; 7 Chemistry Institute, University of São Paulo, São Paulo, Brazil; Institut Pasteur, France

## Abstract

Thymic CD4+CD25+ cells play an important role in immune regulation and are continuously developed in the thymus as an independent lineage. How these cells are generated, what are their multiple pathways of suppressive activity and which are their specific markers are questions that remain unanswered. To identify molecules involved in the function and development of human CD4+CD25+ T regulatory cells we targeted thymic CD4+CD25+ cells by peptide phage display. A phage library containing random peptides was screened ex vivo for binding to human thymic CD4+CD25+ T cells. After four rounds of selection on CD4+CD25+ enriched populations of thymocytes, we sequenced several phage displayed peptides and selected one with identity to the Vitamin D Receptor (VDR). We confirmed the binding of the VDR phage to active Vitamin D in vitro, as well as the higher expression of VDR in CD4+CD25+ cells. We suggest that differential expression of VDR on natural Tregs may be related to the relevance of Vitamin D in function and ontogeny of these cells.

## Introduction

The cells exiting the thymus with the CD4+CD25+ phenotype are known as natural regulatory T cells (Treg) and develop as a continuous and independent lineage in this organ [1], [2]. Thymic development of natural Treg involves the participation of interleukin (IL) -2, CD28, CTLA-4 (cytotoxic T-lymphocyte antigen-4, CD152), and transforming growth factor beta (TGFβ), as well as the expression of the transcription factor Foxp3 (forkhead box P3) [3]. Several other molecules, however, may also play relevant roles.

Natural Tregs actively maintain immunological self-tolerance and control inflammatory immune responses on both antigen presenting cells (APC) [4] and effector T cells [5]. Suppression by regulatory T cells was first shown by the finding that depletion of CD4+CD25+T cells from wild-type mice led to the spontaneous development of several autoimmune diseases, such as autoimmune gastritis, thyroiditis and type 1 diabetes, as well as inflammatory bowel disease [6]. The same group also observed that reconstitution of these animals with CD4+CD25+T cells prevented the development of these diseases. Other groups have shown the importance of natural Tregs in several pathological contexts [7], [8].

Natural Tregs are characterized by the constitutive expression of markers that include the glucocorticoid-induced TNF receptor family–related protein (GITR), OX40 (CD134), CD27 and CTLA-4, CD62-L, and membrane TGFβ [9] [10]. In addition, the transcription factor Foxp3, a critical component of Treg function, can be used to identify these populations [11]. However, all of these markers can also be detected in induced Treg types including T regulatory type 1 (Tr1) [12] and T helper type 3 cells (Th3) [13]. Moreover, non-regulatory T cells can express several of these proteins, and activated human T cells transiently express Foxp3 [14]. Therefore, it is currently not possible to identify and isolate Tregs by a single specific marker. More recently, a transcription factor from the Ikaros family, Helios, was reported to be selectively expressed on natural Tregs and not in induced Tregs, in both mice and humans [15].

Despite the variety of immune active molecules on natural Tregs, indicative of the diversity of mechanisms involved in their suppressive activity, it is likely that several other pathways participate in both the development and function of Tregs. To identify other proteins involved in the function and development of human CD4+CD25+ T regulatory cells, we targeted thymic CD4+CD25+ cells with a peptide phage-display library, using a technology that has allowed the identification of relevant functional molecules in other contexts. Using this approach, we identified a phage-displayed peptide with sequence similarity to the Vitamin D Receptor (VDR) that specifically bound active Vitamin D in vitro. We also showed that CD4+CD25+ express higher levels of VDR than CD25− counterparts, which probably leads to higher binding and signaling by Vitamin D in the first cells. These data support a role for Vitamin D in the development and/or function of CD4+CD25+ human thymocytes.

## Materials and Methods

### Study subjects

Thymic specimens were obtained from children who underwent corrective cardiac surgery at Heart Institute of São Paulo, Brazil. Thymuses were removed only under medical indication, and the children's parents signed the Informed Consent authorizing the use of thymic tissue, which otherwise would have been discarded. The underlying diseases were all congenic; no child had autoimmune diseases. The children's ages ranged from 15 days to 8 years old; 1 was female and 3 were male. This study was approved by the Hospital das Clínicas HCFMUSP Ethical Committee (CAPPesq 404/03).

### Isolation of CD4+ thymocytes and sorting of CD4+CD25+ cells

Human thymic specimens were used as sources of CD4+CD25+ thymocytes. To obtain CD4+ thymocytes we first performed negative selection of CD8+ and double-positive (DP) thymocytes by magnetic cell sorting (MACS - Mylteni Biotec, Germany), as described by the manufacturer. Briefly, thymic mononuclear cell (MNC) suspensions were incubated for 20 min with a mixture of anti-CD14, anti-CD16, anti-CD19, anti-CD34, anti-CD56, anti-glycophorin A, B and anti-CD8 monoclonal antibodies (mAbs), extensively washed, and subsequently incubated for additional 20 min with goat anti-mouse polyclonal Ab conjugated to colloidal super-paramagnetic microbeads, as described in the MACS system protocol. Washed cells were separated on a medium-sized magnetic column (MS+). The purified whole CD4+ thymocyte enriched population was used for the sorting of CD4+CD25+ and CD4+CD25− cells in a high pressure FACSAria cell sorter (BD Biosciences – Mountain View, CA).

### Phage screening

We used a random peptide library comprised of CX_7_C (C, cysteine; X, any amino acid), which was introduced into the fUSE5 vetor digested with Sfi restriction enzyme. X residues are encoded by NNK. The library had a diversity of approximately 2×10^8^ peptides [16]. Screening of the phage library was performed by the BRASIL method [17], which allows the separation of cell-bound from unbound phage in one step. CD4+CD25− cells were used for preclearing and CD4+CD25+ thymocytes were used for biopanning and selection of bound phage.

The cell suspension was incubated with the phage library (10^9^ Transducing units, TU) at 4°C for 2 h. Unbound phages were removed by centrifugation into the oil phase whereas bound phages were recovered from the cell pellet and rescued by addition of K91 host bacteria. The phage output was determined by counting of the number of colonies on agar plates. The eluted phages were propagated in bacterial cells and were used for the next round of selection. After four rounds, individual clones were randomly picked and cryopreserved.

Thymocytes negative for CD25 were initially incubated with the phage library for a subtractive screening; phage clones that did not bind to CD25− cells were further incubated with CD4+CD25+ thymocytes. Four rounds of these negative and positive selections were performed using the BRASIL method, as described above. From each round, the bound phage were retrieved and amplified in K91 *E. coli* for use in the subsequent round of panning.

The DNA inserts of selected phage clones were sequenced on an automatic sequencer (Applied Biosistems). The translated peptide sequences were aligned using Clustal W program (www.expasy.ch) and analyzed with the NCBI BLAST (http://www.ncbi.nlm.nih.gov) to identify proteins with similar motifs.

### Binding assays of phage clones to cells

Thymocytes were separated using a FACSAria and were ressuspended in Dubelcco Modified Elementary Media (DMEM) containing 1% bovine serum albumin (BSA). The cell suspension was incubated with the amplified target phage or with a control phage having no inserted peptide (Fd phage) and the mixture was kept on ice for 4 h. Cells were again separated by centrifugation through an organic oil phase, and bound phages were recovered by bacterial infection and quantified by colony count.

### Binding assays of phage clones to proteins using ELISA

1,25-dihydroxy vitamin D3 (1 mM) (from now on called Vitamin D), vitamin D receptor (VDR) (200 uM) (Fluka Biochemika, St. Gallen, Switzerland) or BSA (3% in Phophate Buffer Solution- PBS) were immobilized overnight on high-bind 96 TM-well plates (Pierce/Thermo Fisher- Waltham, MA) at 4°C. The wells were washed twice with PBS, blocked with PBS containing 3% BSA at room temperature for 2 h, and incubated with 1×10^9^ TU of VDR phage, negative control Fd phage or VDR phage, previously incubated with Vitamin D in 50 µl of PBS containing 1.5% BSA. After 1 h at room temperature, wells were washed 9 times with PBS, and phage binding was measured by enzyme-linked immuno sorbent assay (ELISA). Results were calculated subtracting the OD from phage bound to BSA.

### Flow cytometry analysis

Intracellular expression of both VDR and Foxp3 was evaluated in thymocytes by flow cytometry (FACSCanto, BD Biosciences, Palo Alto, CA), and analyzed using FlowJo (TreeStar Inc). Cells were stained by anti-CD4PerCP or anti-CD4FITC, anti-CD25APC or anti-CD25PE and anti-CD8 Texas Red, then fixed and permeabilized before staining with anti-Foxp3PE and anti-VDR followed by secondary anti –antibody APC-labeled. All mAbs were purchased from BD Biosciences except anti-Foxp3 and secondary APC, provided by eBioscience and anti-VDR, provided by Affinity Bioreagents.

### Statistical analysis

All samples from the three ELISA binding assays were analyzed using Kolmogorov-Smirnov and D'Agostino-Pearson normality tests, and both indicated non-parametric distribution. Kruskal Wallis was then used to compare data from the four independent groups, obtained after subtraction of BSA binding ODs. Significant statistical difference was considered when p<0.05.

## Results

### CD4+CD25+ thymic cell isolation

In order to separate CD4+CD25+ from CD4+CD25− thymocytes we performed cell sorting by high-pressure flow cytometry in a FACSAria sorter. Different numbers of thymocytes were used for the 4 rounds of selection with the phage library. The gate strategy for the sorting of CD4+CD25+ cells is shown in figure S1. The following enrichment of CD4+CD25+ cells was achieved: 44.4% (2×10^6^ cells), 57.3% (9.7×10^5^ cells), 39.5% (1.4×10^6^ cells), and 72.7% (5.4×10^6^ cells), respectively, for rounds 1 to 4. Despite the relatively low purity of the selected CD4CD25+ population, the strategy of pre-clearing (subtraction) in a 90% pure CD4+CD25− population reinforced the selection for phages that bound preferentially to CD25+ cells.

### Selection of phage-binding CD4+CD25+ thymic cells

**Figure 1:**
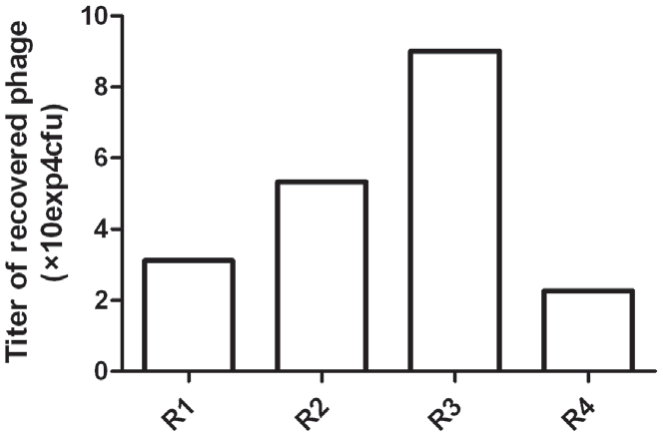
Specific enrichment of CD4+CD25+ cell–bound phages. The titer of phages recovered from each round was evaluated by a plaque-forming assay. R1–R4, 1st–4th round of panning in vitro. Cfu: colony forming units.

The phage clones that did not bind to the CD25 negative cells were incubated with the CD4+CD25+ thymocytes to select phages that bind specifically to the CD25 positive population. The number of phages recovered from the second incubation with CD4+CD25+ cells increased 1.5 fold over the number of phages recovered from round 1 (Figure 1 and Table 1). The output-input ratio of phage after each round of panning was used to determine the efficiency of phage recovery [18]. We observed a 3-fold enrichment from the first to the third round (output/input from 6 to 18×10^−6^). In the fourth round, in which we used the most enriched CD4+CD25+ population for panning, the enrichment decreased compared to third round (Table 1), probably indicating a more stringent selection compared to the other rounds.

**Table 1:** Output/input values for thymic CD4+CD25+ selection.

Round of Panning	Input Number	Output Number	Ratio
1	5×10∧9	3×10∧4	6×10∧-6
2	5×10∧9	5×10∧4	10×10∧-6
3	5×10∧9	9×10∧4	18×10∧6
4	5×10∧9	2×10∧4	4×10∧6

The ratio was calculated as output number/input number of phages (cfu).

cfu: colony forming units.

### Candidate phage displays similarity to the Vitamin D Receptor

After 4 rounds of panning of CD4+CD25+ thymocytes with the phage library, approximately 700 clones were picked and cryopreserved and 28 phages were chosen at random. The peptide-coding DNA inserts of the 28 phage clones were amplified by PCR and sequenced. Alignment of peptide sequences using Clustal W program revealed no repeated sequences among the selected phages (Table 2). A list of the selected phage peptides and their corresponding human proteins is presented in Table 2. Among the identified bound phage peptides we found proteins known to be Treg markers, such as LAP, GITR, IL12p25 (subdomain of IL35, a Treg hallmark cytokine), which validates our approach.

**Table 2:** Selected peptide sequences and the corresponding human proteins containing the homologous motif.

Peptide	Motif	Protein	Accesion Number
CLLGTRWPC	**LLG**A**RW**FPKTL**PC**	toll-like receptor 7 [Homo sapiens]	gi|76780835
CFMESVGRC	**FME**G**VG**	leptin receptor [Homo sapiens]	gi|1589772
CLTPEFHIC	**C**V**TPE**Y**H**CGDPQCKIC	tumor necrosis factor receptor superfamily member 18 isoforma 2 (GITR-D, GITR-C e GITR-B) (Homo sapiens)	gi|23238194
CLAVGEVLC	**LAV**A**E**C**LC**	interleukin 17C [Mus musculus]	gi|22003880
CFVSPPVGC	**CF**C**SPP**	Latent transforming growth factor beta-binding protein 2	gi|62089316
CMPGWEVLC	**PGWEV**	IL-13p600 [Homo sapiens]	gi|19070472
CKRGNSGSC	**NSGSC**	semaphorin 5B isoform 2 [Homo sapiens]	gi|72534694
CQRLVGFAC	**CQRLVG—FA**	thyroid hormone receptor-associated protein complex component TRAP240 [Homo sapiens]	gi|4530437
	**CQRL**MTF	Toll-like receptor 6 [Mus musculus]	gi|33286898
	**Q**KVV**GFA**	vitamin D receptor [Homo sapiens]	gi|4262865
CLQASPNFC	**CIQA**G**PN**	Integrin (CD29) [Homo sapiens]	
	**CLQ**S**S**G**NF**	E-selectin (ELAM-1) [Homo sapiens]	gi|462500
CNGSVRSFC	**NGS**C**R**D**FC**	defensin, beta 108B [Homo sapiens]	gi|50344744
	**GSV**G**SFC**	transforming growth factor, beta 2 [Rattus norvegicus]	gi|13592109
CPGFGLAYC	**GFGL**PY	latent TGF-beta binding protein-4 [Homo sapiens]	gi|2190402
	**GFGLA**F	interleukin 20 [Mus musculus]	gi|84627505
	**PGFGL**	interleukin 12 p35 subunit [Sus scrofa]	gi|47522812
CFLFTFEAC	**CFLFT**	similar to 60 kDa heat shock protein, mitochondrial precursor (Hsp60) [Bos taurus]	gi|76648520
CRGVLMRYC	**MRYC**	semaphorin 5A [Homo sapiens]	gi|4506881
CRAFVVASC	**CR**S**F**A**VA**I**C**	C9 complement protein[Homo sapiens]	gi|6706618
CQSHSAFVC	**SAF**S**C**	interleukin-21 [Homo sapiens]	gi|11141875

Peptides were analyzed with the NCBI BLAST search against the SWISSPROT database, with the option for short nearly exact matches.

**Figure 2:**
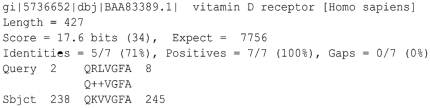
BLAST hit for VDR phage. Length: of the Vitamin D Receptor; Score: punctuation score given for the alignment; Expected: e-value; Identities: number of identical amino acids between queried sequence and the corresponding amino acid sequence of the VDR protein; Positives: number of amino acids that align to the VDR protein; Gaps: spaces between amino acids that do not match; Query: VDR phage peptide sequence; Sbjct: fragment of the VDR that matches to the phage sequence. The numbers indicate the position of the amino acids in the phage peptide and in the VDR protein.

**Figure 3:**
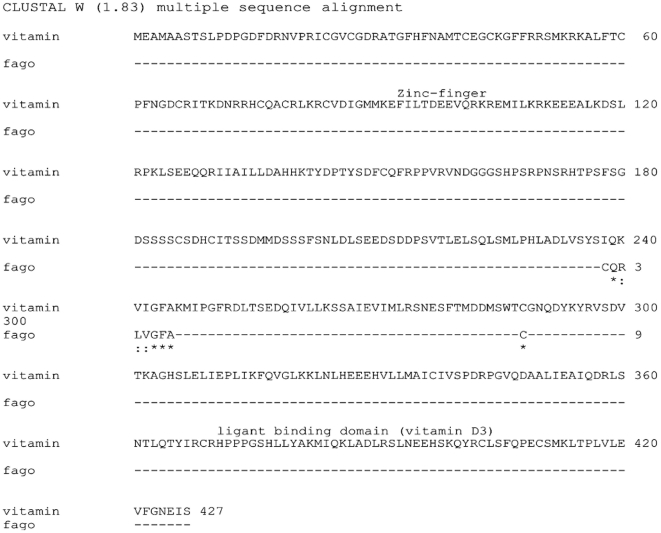
ClustaW alignment of the VDR phage peptide sequence and the human VDR full-length protein sequence.

The Vitamin D receptor phage (VDR phage) peptide showed similarity to the Vitamin D_3_ receptor. Five amino acids (aa) exhibited total identity with the VDR sequence and two aa were conservative substitutions. In Figure 2 we show the vitamin D hit obtained following BLAST analysis. To identify the region of the VDR that matches the VDR phage, we aligned the full-length protein sequence of the VDR with the phage peptide (Figure 3). The VDR phage peptide aligned with the region of the VDR that interacts with the Vitamin D ligand (aa 239–246).

**Figure 4:**
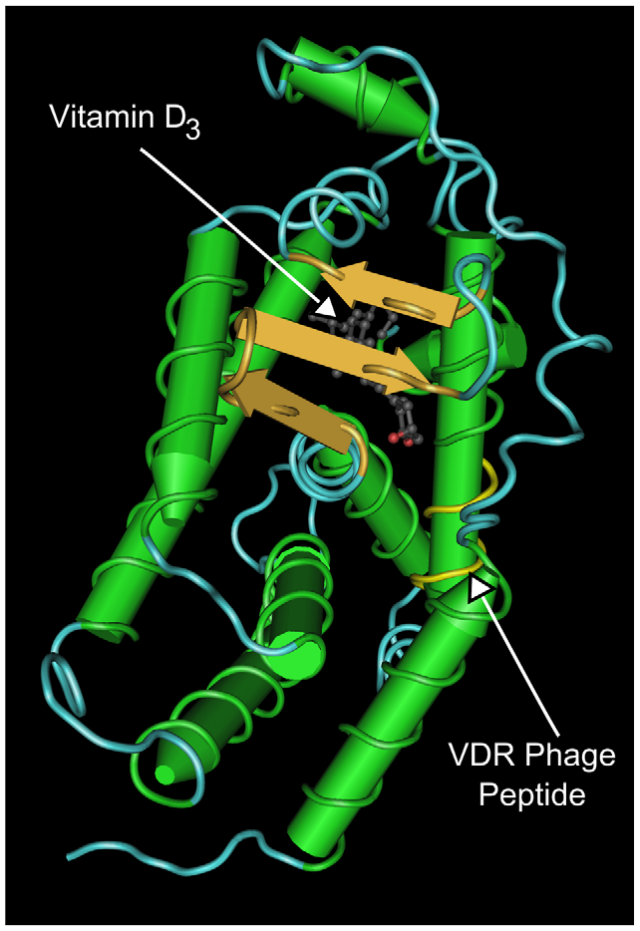
Structural model of the Vitamin D Receptor associated with its ligand (Vitamin D). Green: alpha helical sequences; gold: beta sheet; blue: structural handle, yellow: phage peptide.

Using the RasMol program (http://www.ncbi.nlm.nih.gov), we assessed the 3D-structure of the VDR complexed to Vitamin D. The structural model of the receptor revealed that the phage peptide region is located in one of the alpha helices that compose the ligand binding domain. The position of the VDR phage peptide within the structure of the VDR is shown in Figure 4.

### VDR phage binds to CD4+CD25+ thymocytes and to immobilized active Vitamin D3

**Figure 5:**
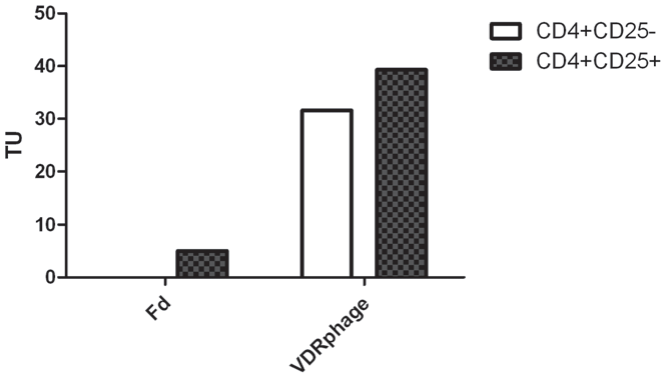
Binding assay with thymic CD4+CD25+ cells and CD4+CD25− cells. Fd: wild type phage bearing no inserted peptide sequence; TU: transforming units.

To analyze the binding specificity of VDR phage to CD4+CD25+ thymocytes we performed a comparative binding assay with CD4+CD25+ as well as CD4+CD25− cells. A higher binding of the VDR phage to CD4+CD25+ cells was observed in this experiment, as shown in Figure 5.

**Figure 6:**
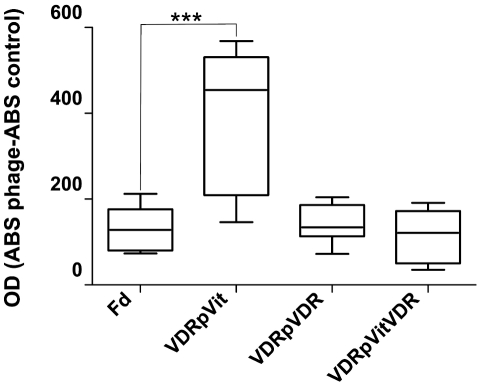
ELISA to evaluate the binding of the VDR phage to plate-bound Vitamin D or VDR. Fd shows the binding of the control phage to plate-bound Vitamin D; VDRpVit shows the binding of the VDR phage to plate-bound Vitamin D; VDRpVDR shows the binding of the VDR phage to plate-bound VDR; VDRpVitVDR shows binding of the VDR phage pre-incubated with Vitamin D to plate-bound VDR. *** p = 0.001.

To validate the ligation of the VDR phage to Vitamin D, an ELISA assay was performed with immobilized Vitamin D or VDR. The targeted VDR phage bound more to active Vitamin D than an “insertless” control phage (Fd phage, Figure 6), indicating that VDR peptide is responsible for binding to Vitamin D. Preincubation of VDR phage with Vitamin D prevented the ligation of Vitamin D to its active receptor, confirming that the peptide corresponds to Vitamin D binding pocket of VDR molecule.

### Higher VDR expression in CD4+CD25+ thymocytes and in Foxp3+ thymocytes

**Figure 7:**
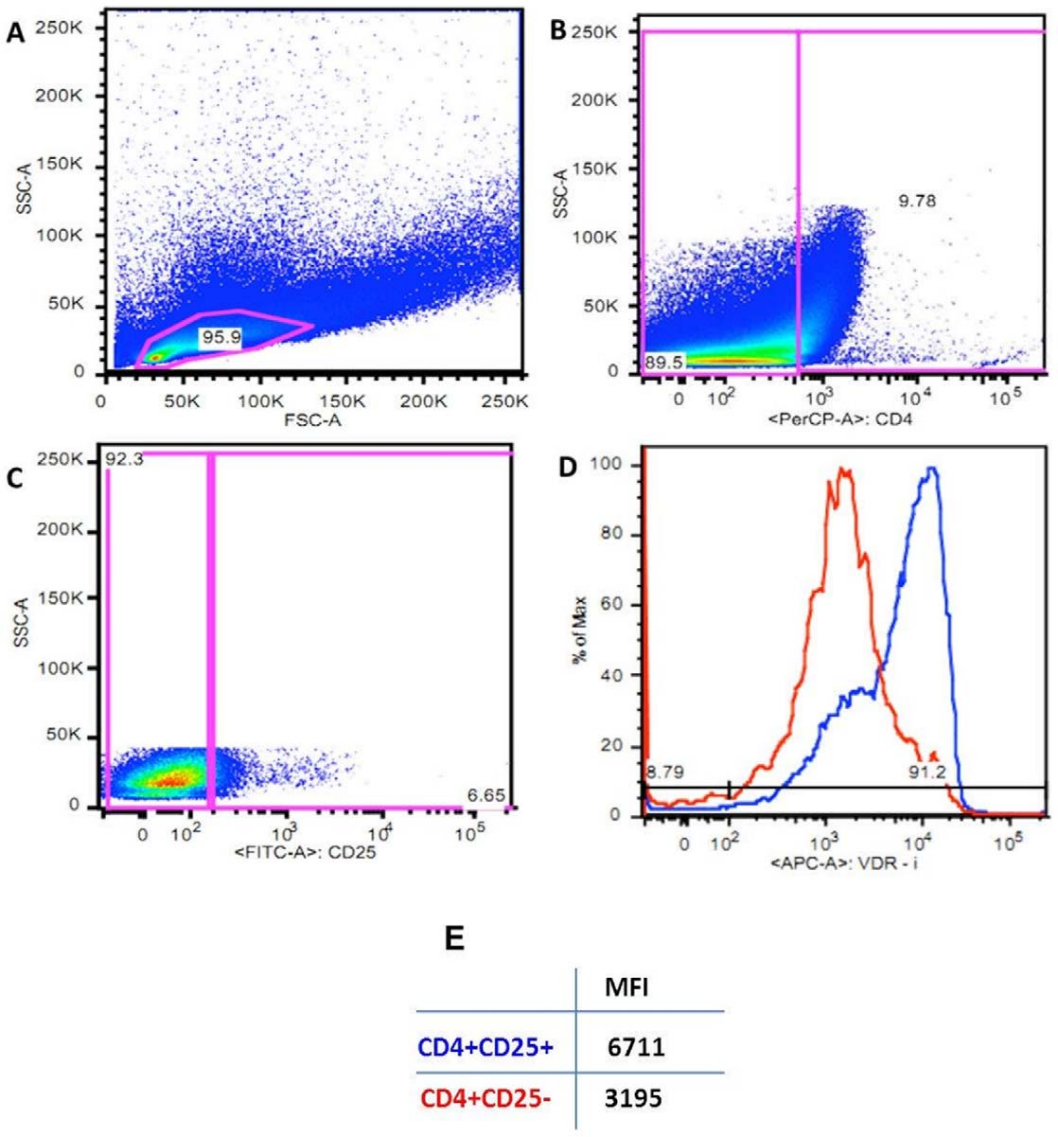
FACS analysis of VDR expression in CD25+ and CD25− cells in the CD4+ cell compartment. A: Dot plot showing thymocytes region; B: Dot plot showing the CD4+ gated cells; C: Dot plot showing percentage of CD25positive and negative cells in the CD4+ region; D: Histogram showing the expression of VDR in CD4+CD25+ (blue) and CD4+CD25−(red) thymocytes; E: Table showing MFI (median fluorescence intensity) of the intracellular VDR labeling of the cells.

To verify whether the enhanced binding of the VDR phage to CD4+CD25+ cells in comparison to CD4+CD25− cells was related to a higher expression of VDR in CD25 positive cells, we analyzed the expression of the VDR in thymocytes by FACS using a VDR specific antibody. VDR expression was 2.5 fold higher in CD4+CD25+ thymocytes than in CD4+CD25− cells (Figure 7), despite being expressed in all thymic cells.

**Figure 8:**
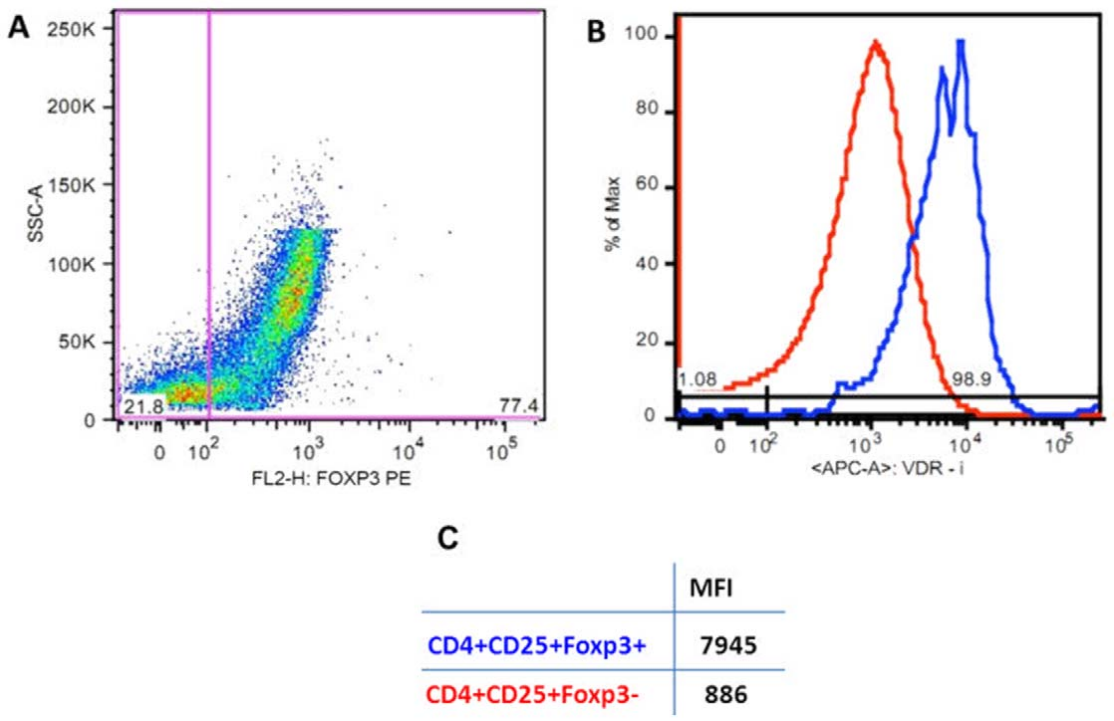
FACS analysis of VDR expression in Foxp3+ and Foxp3− cells in the CD4+CD25+ cell compartment. (Figure 7C region). A: Dot plot showing Foxp3 region in CD4+CD25+ thymocytes; B: Histogram showing the expression of VDR in CD4+CD25+Foxp3+ (blue) and CD4+CD25+Foxp3− (red) cells; C: Table showing MFI (median fluorescence intensity) of the intracellular VDR labeling of the cells.

We also analyzed the relation between VDR expression and Foxp3 expression in thymic cells. Again, VDR expression was almost 3 fold higher (2.79 fold) in Foxp3+ CD4+CD25+ thymocytes (Figure 8). All CD25 positive cells in the thymus expressed Foxp3: double negative, double positive, and CD4 or CD8 single cells (Figure S2).

## Discussion

Our aim in this work was to search for proteins expressed on human CD4+CD25+ thymic cells that were potentially relevant to the function and development of natural regulatory T cells.

Phage display technology has been used to identify peptide ligands in different systems and in a variety of cell types *in vitro* and *in vivo* [19]. Although antibody phage display has been useful for the identification of molecules involved in homing to the thymic endothelia [20], it was still unclear whether this technique could be appropriate for the study of new markers in natural Tregs.

In this study we used phage display to define binding sites on nTregs. The phage peptides that bound to Tregs might be exploited as a means to expand current knowledge of nTreg interactions during the development of this subtype of T cells. Besides, once these sites are clearly identified, the peptides may be used to study molecules which could improve or block the regulatory activity.

Using a peptide phage display library in a screening protocol that included subtraction and positive selection on intact viable thymocytes, we identified peptides that preferentially bound to CD4+CD25+ cells. The optimizing procedures with several rounds of panning improved the probability of obtaining phages that preferentially bound to target cells. In fact, in most cases we observed an increase in the output/input ratio during the successive rounds (Figure 1). However, those data should be analyzed considering the CD4+CD25+ thymocyte purity during each step. In the first three rounds, we observed a progressive enrichment of output phages (3 fold increase), while a decrease was seen from the third to the fourth step. This decrease can be attributed to a selection using a specially enriched CD4+CD25 population in the fourth round (72% compared to 39 to 53% in the other rounds). Consequently, phages selected in the first three rounds may include those that bound to CD4+CD25+ and some that bound to CD25− cells, while phages recovered from the fourth round would be preferentially directed to our target CD4+CD25+ population. Besides, the negative selection of phages (pre-clearing) using a 90% pure CD4+CD25− population compensated for the relative low purity of CD4+CD25+ populations used for positive selection (panning).

Among the phages sequenced we selected a specific phage peptide for further analysis due to its sequence similarity to the Vitamin D receptor (VDR). This was our first choice because although Vitamin D has been extensively reported to display immunoregulatory functions in different contexts [21], its role has not been well-defined for human thymic T regs, opening an interesting area for research.

We hypothesized that the ligand recognized by the VDR phage was Vitamin D complexed to its receptor on CD4+CD25+ thymocytes, because the sequence represented by the phage peptide is located in the VDR region that interacts with the Vitamin D ligand (aa 239–246) and the structural model of the receptor revealed that the phage peptide region is located in one of the alpha helices that compose the ligand binding domain (LBD). The results from the ELISA assay showing that VDR phage was able to bind to active Vitamin D strongly supports our hypothesis. When phages were preincubated with Vitamin D we did not observe binding to VDR, confirming that the peptide, in fact, mimic the Vitamin D binding pocket in VDR (Figure 2) and is able to prevent Vitamin D from binding to its native receptor.

We do not know whether the identified peptide is an oligomerization domain. However, it is known that upon activation by vitamin D, VDR usually forms a heterodimer with the retinoid-X receptor and binds to hormone response elements on DNA, resulting in the expression or transrepression of specific gene products. It has also been reported that a predominant C-terminal heterodimerization domain resides between residues 382–403 in VDR sequence, about 137 aa distant from our VDR-phage. It was shown that mutations inserted in this region of the VDR sequence (Lys 382, Met-383 or Glu-385) completely disrupted the associations of VDR-RXR and VDR with other partners, and eliminated the transcriptional activity of VDR [22], indicating the importance of this region to heterodimerization. Other partners can also physiologically dimerize with VDR such as Mediator of RNA polymerase II (MED1 and MED12) [23], nuclear receptor co-repressor (NCOR1) [24] and nuclear receptor co-activator (NCOA2) [25], co-activators or co-repressors that direct transcriptional initiation by the RNA polymerase II apparatus [26].Thus, although the 382–403aa domain in VDR has been shown to be important for dimerization, we cannot exclude the existence of other domains too.

The higher expression of VDR observed in CD25+ compared to CD25− cells (Figure 7), if associated with a corresponding higher binding of Vitamin D to VDR in these cells, supports our isolation of VDR phage after panning in CD4+CD25+ thymocytes. Another important observation was the positive relation between Foxp3 expression and VDR expression in thymic cells (Figure 8). Studies using mice in a Trinitrobenzene Sulfonic Acid (TSA) induced colitis model, treated with Vitamin D and dexamethasone, show that the combined use of Vitamin D and dexamethasone enhanced Foxp3 expression accompanied by the induction of IL-10 and TGF-beta [27]. However, in vitro-generated homogenous populations of IL-10 Tregs obtained by stimulating naive CD4 T cells in the presence of the anti-inflammatory drugs Vitamin D and dexamethasone did not express high levels of Foxp3 [28]. Thus, although Foxp3 appears to be important for the development and function of naturally occurring CD4+CD25+ T cells, in vitro derived IL-10-secreting Tregs appear to have regulatory functions despite low levels of Foxp3.

Here, we report a high expression of VDR in thymic CD25+Foxp3+ suggesting that VDR may, indeed, be involved in the natural regulatory T cell lineage development in the thymus. Interestingly, molecules known to dimerize with VDR such as retinoic X receptor (RXR) have been shown to physically interact with Foxp3 [29]. The complexed VDR can dimerize with Vitamin A receptors (the retinoic acid receptor and the retinoic X receptor) and bind to Vitamin D response elements (VDrEs) in the promoters of Vitamin D-responsive genes [30]. Given that Vitamin A has roles in immunoregulation that include the generation of induced Tregs [31], [32], [33], we hypothesize that Vitamins D and A might act synergistically in immunoregulatory pathways and possibly on natural Tregs.

An interesting question is whether the VDR phage ligand on CD4+CD25+ thymocytes is located intracellularly or on the cell surface. Although it is generally accepted that peptides presented by phage particles bind to surface molecules, investigators have reported that some phage can reach intracellular compartments by an yet unknown mechanism [9]. Taking into consideration that phage can be readily internalized by cells [34], [35], it is thus possible that the VDR phage binds directly to Vitamin D inside the cell. We did observe higher VDR intracellular expression in CD4+CD25+ thymocytes in comparison to CD4+CD25− cells (Figure 7), but we were unable to detect VDR cell surface expression using the same commercial anti-VDR antibody (data not shown). We cannot be sure, however, that the anti-VDR antibody we used in our study is appropriate for cell surface expression. Nevertheless, the VDR could also (or alternatively) be expressed at the cell surface, as suggested by others [36], [37], [38].

Taken together, our results favor that Vitamin D has functions in natural Treg activity or ontogeny. Although the role of Vitamin D in natural Tregs immunobiology needs to be better defined, data from several groups showing its immunoregulatory activity on different cells [39] reinforce the idea that similar functions may also be relevant for natural Tregs. The immunoregulatory role of Vitamin D has been studied in the induction of tolerogenic dendritic cells with the production of IL-10 and TGF-beta [40], [41] and in the generation of induced Tregs [42]. However, in the majority of studies on T cells, Vitamin D was used in combination with other molecules, such as dexamethasone [43] and IL-2 [44]. More recently, Vitamin D has been reported to induce and/or increase the expression of Foxp3 and CTLA-4 in purified CD4+CD25− human T cells [44]. It was also suggested that Vitamin D interacts with Vitamin D Responsive Elements (VDRE) in the Foxp3 gene and enhances Foxp3 expression in CD4+ cells [29]. Other data that support an immunoregulatory role for Vitamin D are derived from animal models of autoimmune diseases, in which the use of Vitamin D partially suppresses the development of experimental autoimmune encephalomyelitis, inflammatory bowel disease, and diabetes [45], [46], [47]. The mechanisms by which Vitamin D reduces the inflammatory process in these contexts remain unclear.

In summary, our data support the notion of an immunoregulatory role for Vitamin D in natural Tregs and a functional relationship between Foxp3 and VDR in the differentiation of these cells in the thymus. This observation may shed light on possible interventional strategies for Tregs, contributing to this ongoing rich research field.

## Supporting Information

Figure S1 **FACS Dot Plot graph showing the percentage of Foxp3 population in thymus.** A: Dot Plot showing the lymphocyte region; B: FACS Dot Plot showing the region of double positive cells; C FACS Dot Plot showing the region of double negative cells; D: FACS Dot Plot showing the region of CD8 single positive cells; E FACS Dot Plot showing the region of CD4 single positive cells. From B to E: Left: Dot Plot; Central: Histogram of Foxp3 staining. Right: Table showing MFI (median fluorescence intensity) of the intracellular Foxp3 labeling of the cells.
(TIF)

Figure S2 **FACS Dot Plot graph showing percentage of enriched CD4+CD25+ population used for phage peptide screening.** Example from the fourth round of panning. A: Dot Plot showing the lymphocyte region gated for CD4+ enriched cell sorting; B: FACS Dot Plot showing the region selected for CD4+ sorting; C and D: FACS Dot Plot showing the percentage of thymocytes after sorting; C: CD4+CD25− thymocytes and D: CD4+CD25+ thymocytes.
(TIF)
