# Supplementary figures and images for: Identification of Novel Immunoregulatory Molecules in Human Thymic Regulatory CD4+CD25+ T Cells by Phage Display
Source: PLoS One. 2011 Aug 1;6(8):e21702. doi: 10.1371/journal.pone.0021702 (PMC3148221; doi:10.1371/journal.pone.0021702)

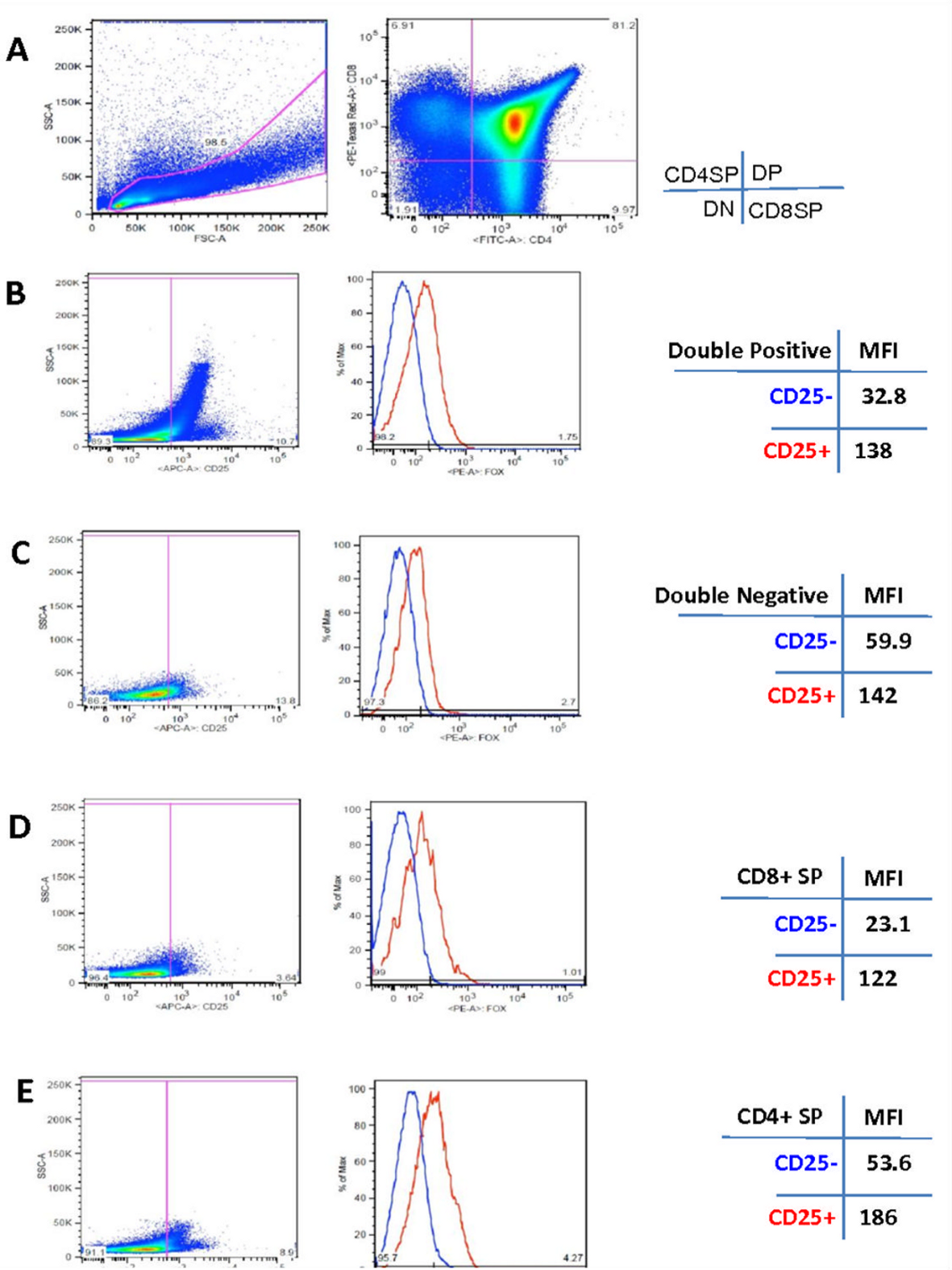

Supplement: Figure S1 — FACS Dot Plot graph showing the percentage of Foxp3 population in thymus. A: Dot Plot showing the lymphocyte region; B: FACS Dot Plot showing the region of double positive cells; C FACS Dot Plot showing the region of double negative cells; D: FACS Dot Plot showing the region of CD8 single positive cells; E FACS Dot Plot showing the region of CD4 single positive cells. From B to E: Left: Dot Plot; Central: Histogram of Foxp3 staining. Right: Table showing MFI (median fluorescence intensity) of the intracellular Foxp3 labeling of the cells. (TIF) [file pone.0021702.s001.tif]

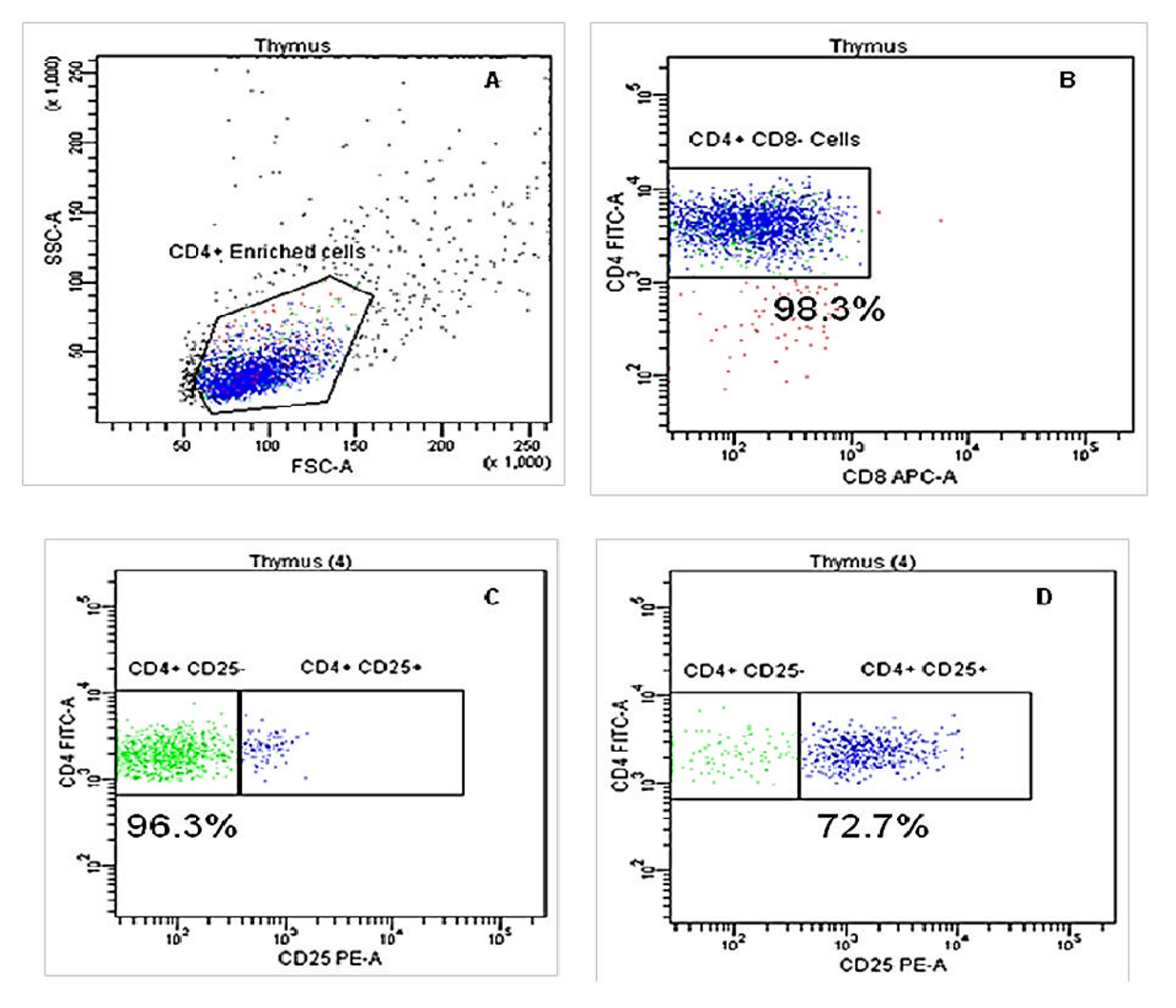

Supplement: Figure S2 — FACS Dot Plot graph showing percentage of enriched CD4+CD25+ population used for phage peptide screening. Example from the fourth round of panning. A: Dot Plot showing the lymphocyte region gated for CD4+ enriched cell sorting; B: FACS Dot Plot showing the region selected for CD4+ sorting; C and D: FACS Dot Plot showing the percentage of thymocytes after sorting; C: CD4+CD25− thymocytes and D: CD4+CD25+ thymocytes. (TIF) [file pone.0021702.s002.tif]
